# Survival Analysis and Contributing Factors among PCR-Confirmed Adult Inpatients during the Endemic Phase of COVID-19

**DOI:** 10.3390/diseases11030119

**Published:** 2023-09-11

**Authors:** Verónica Benites-Godínez, Oliver Mendoza-Cano, Xóchitl Trujillo, Mónica Ríos-Silva, Agustin Lugo-Radillo, Jaime Alberto Bricio-Barrios, Herguin Benjamin Cuevas-Arellano, Eder Fernando Ríos-Bracamontes, Walter Serrano-Moreno, Yolitzy Cárdenas, Greta Mariana Baltazar-Rodríguez, Ana Daniela Ortega-Ramírez, Efrén Murillo-Zamora

**Affiliations:** 1 Coordinación de Educación en Salud, Instituto Mexicano del Seguro Social, Calzada del Ejercito Nacional 14, Tepic 63169, Mexico; 2 Unidad Académica de Medicina, Universidad Autónoma de Nayarit, Ciudad de la Cultura Amado Nervo, Tepic 63155, Mexico; 3 Facultad de Ingeniería Civil, Universidad de Colima, km. 9 Carretera Colima-Coquimatlán, Coquimatlán 28400, Mexico; 4 Centro de Estudios e Investigación en Biocultura, Agroecología, Ambiente y Salud Colima, Ex-Hacienda Nogueras S/N, Nogueras 28450, Mexico; 5 Centro Universitario de Investigaciones Biomédicas, Universidad de Colima, Av. 25 de Julio 965, Colima 28045, Mexico; 6 Centro Universitario de Investigaciones Biomédicas, CONAHCyT—Universidad de Colima, Av. 25 de Julio 965, Colima 28045, Mexico; 7 CONAHCyT—Faculty of Medicine and Surgery, Universidad Autónoma Benito Juárez de Oaxaca, Ex Hacienda Aguilera S/N, Carr. a San Felipe del Agua, Oaxaca 68020, Mexico; 8 Facultad de Medicina, Universidad de Colima, Av. Universidad 333, Colima 28040, Mexico; 9 Facultad de Ciencias, Universidad de Colima, Bernal Díaz del Castillo 340, Colima 28045, Mexico; 10 Departamento de Medicina Interna, Hospital General de Zona No. 1, Instituto Mexicano del Seguro Social, Av. Lapislázuli 250, Villa de Álvarez 28984, Mexico; 11 Escuela de Medicina y Ciencias de la Salud, Instituto Tecnológico y de Estudios Superiores de Monterrey, Campus Guadalajara, Av. General Ramón Corona No. 2514, Zapopan 45201, Mexico; 12 Programa de Doctorado en Ciencias Médicas, Facultad de Medicina, Universidad de Colima, Av. Universidad 333, Colima 28040, Mexico; 13 Unidad de Investigación en Epidemiología Clínica, Instituto Mexicano del Seguro Social, Av. Lapislázuli 250, Villa de Álvarez 28984, Mexico

**Keywords:** COVID-19, survival rate, inpatients, polymerase chain reaction

## Abstract

In May 2023, the global health emergency status of COVID-19 concluded, marking the onset of an endemic era. This study assessed survival rates among PCR-confirmed adult inpatients during this phase and determined contributing factors. Employing a survival analysis approach, this investigation utilized a nationwide Mexican cohort encompassing 152 adult inpatients. Survival rates were computed using the Kaplan–Meier method, and a proportional Cox model identified mortality risk factors. Survival rates remained above 65% on day 14 after admission. Vaccination status, including the number of doses administered, was not significantly associated with fatal outcomes. Chronic kidney disease or a history of immunosuppression (due to any cause) increased mortality risk. Our findings underscore the persistent severity of COVID-19 beyond the global health emergency, emphasizing the necessity for tailored interventions for vulnerable patients.

## 1. Introduction

The emergence of the coronavirus disease 2019 (COVID-19) pandemic, resulting from severe acute respiratory syndrome coronavirus 2 (SARS-CoV-2) infection, necessitated immediate and concerted cooperation among healthcare systems, the scientific community, and governmental bodies to effectively address the repercussions of the infection on public health [1]. Progressing alongside the advancement of vaccines, substantial headway was achieved in curtailing the transmission and mitigating the virulence of the virus [2]. Consequently, the designation of COVID-19 as a global health emergency was lifted, and the disease transitioned into an endemic state [3].

In Mexico, the COVID-19 pandemic became a major social and economic burden. Nonetheless, since the declaration of the end of the global emergency, there have been notable reductions in hospitalization rates, as well as diminished morbidity and mortality [4].

While attention has been largely focused on acute management, transmission control, and vaccine deployment during the pandemic’s peak, the transition from a global health emergency to a post-emergency phase warrants a thorough investigation [5]. This transition not only signifies a pivotal milestone in pandemic management but also presents an opportunity to comprehend the long-term implications of COVID-19 for individuals who have experienced the disease [6]. Understanding the survival outcomes and health trajectories of adult inpatients who tested positive for COVID-19 can provide insights into the lingering effects of the virus and inform healthcare strategies in the post-pandemic era.

This study aims to evaluate the survival experience of adult inpatients who were diagnosed with PCR-confirmed COVID-19 after the conclusion of the global health emergency. Therefore, the investigation aims to contribute to the growing body of knowledge concerning the dynamics of COVID-19 as it transitions from an acute health crisis to a manageable endemic condition. This study intends to provide valuable insights that can guide healthcare policies, clinical decision making, and public health strategies in the aftermath of the global health emergency.

## 2. Materials and Methods

We conducted a nationwide cohort study in Mexico, starting at the outset of the COVID-19 pandemic, which emerged in late February 2020. A more comprehensive description of the cohort has been previously published [7]. This specific investigation, characterized as a survival analysis study, centered on a subset of adult patients aged 18 years and above, presenting respiratory symptoms indicative of COVID-19 between 5 May and 26 July 2023. The inclusion criteria involved confirming disease through RT-PCR testing. Patients who did not necessitate hospitalization were omitted, as well as those with incomplete clinical and epidemiological data of interest.

Patients were identified using the records of a system for the epidemiological surveillance of viral respiratory diseases, which specifically targets SARS-CoV-2, influenza virus, respiratory syncytial virus, and other pathogens of public health concern. This system is known as SINOLAVE, and it primarily derives data from patients’ medical records and, when applicable, death certificates [8].

Demographic and clinical variables of interest were derived from the audited surveillance system. We employed pneumonia at the time of hospital admission as an indicator of severity. This was defined by the presence of respiratory clinical symptoms (such as cough, fever, dyspnea, and chest pain), as well as radiographic evidence of pneumonia (bilateral ground glass opacities or consolidations visible on CT or X-ray).

Nucleic acids were extracted from 200 μL of clinical specimens (deep nasal swabs) using the MagNa Pure LC Total Nucleic Acid Isolation Kit automated system (catalog: 03038505001; Roche Diagnostics, Mannheim, Germany) [9]. The detection of SARS-CoV-2 was carried out utilizing the primers and probes proposed by Corman et al. [10], employing the SuperScript III Platinum One-step qRT-PCR System (catalog: 12574035; Invitrogen, Carlsbad, CA, USA) in conjunction with the 7500 Fast Real-Time PCR System (Applied Biosystems, Foster City, CA, USA).

For the analysis of hospitalized patients with molecularly diagnosed COVID-19, we applied the Kaplan–Meier method to compute survival functions and determine 95% confidence intervals (CIs). The primary binary outcome was in-hospital death due to any immediate cause in patients with PCR-confirmed COVID-19, serving as the failure event. Patients who were still hospitalized as of 5 August 2023 (the date of data compilation) were considered censored observations. To assess the factors associated with patient survival, a multiple proportional Cox hazard regression model was employed.

This study underwent review and approval by the Local Committee of Ethics in Health Research (601) of the IMSS (approval R-2022-601-022). None of the participants were physically located or interviewed during any stage of this study, and all researchers adhered to strict ethical guidelines.

## 3. Results

Data from 152 inpatients with laboratory-confirmed COVID-19 were analyzed, resulting in a total follow-up period of 1143 person-days. They represented 6.2% (*n* = 152/2441) of the total laboratory-positive patients registered during the study period. The remaining cases (93%, *n* = 2289/2441) were patients with mild symptoms who did not require hospital admission and were therefore excluded.

We documented a total of 25 deaths, as indicated by the discharge information present in medical records and subsequently validated by death certificates. Thus, the overall mortality rate was calculated as 2.2 per 100 person-days (25 deaths over 1143 person-days). The median survival time (and the interquartile range) was 6 (4–9) days among cases resulting in a fatal outcome. For patients who successfully recovered, the median duration of in-hospital stay was 6 (3–11) days. No statistically significant differences (*p* = 0.615) were observed between these latter two groups.

As presented in Table 1, the participants who experienced a fatal in-hospital outcome were more likely to exhibit pneumonia at the time of hospital admission (40.0% vs. 14.2%, *p* = 0.002). They also had a higher prevalence of a personal history of type 2 diabetes mellitus (52.0% vs. 20.5%, *p* = 0.001), chronic kidney disease (CKD; 28.0% vs. 6.3%, *p* = 0.001), and immunosuppression due to any cause (20.0% vs. 1.6%, *p* < 0.001). No significant differences were observed in terms of disease outcomes in relation to COVID-19 vaccination status, gender, age, or other clinical characteristics.

The average time span between the date of the final vaccine administration and the onset of symptoms was 504.4 ± 221.1 days for individuals who had received two doses and 445.9 ± 196.9 days for those who had received a vaccine booster (three doses). The lone patient within the study cohort who had received only one vaccine shot encountered symptom onset 463 days after being vaccinated.

**Figure 1:**
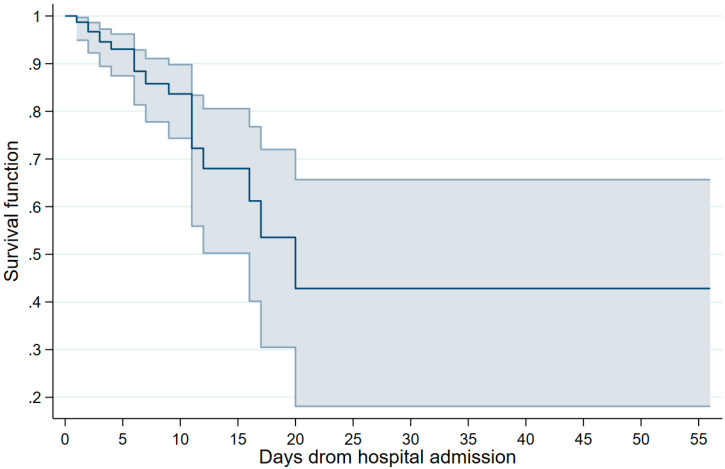
Survival curve of analyzed patients, Mexico 2023. Note: the point estimates and 95% confidence intervals are presented.

The survival rates are summarized in Figure 1. The estimates, along with their corresponding 95% CIs, were as follows, based on the days elapsed since hospital admission: 1 day, 0.98 (0.96–0.99); 3 days, 0.94 (0.91–0.96); 7 days, 0.84 (0.80–0.88); 14 days, 0.66 (0.56–0.73); 21 days, 0.56 (0.43–0.66); and 28 days, 0.47 (0.31–0.61).

In the multiple proportional hazards model (Table 2), the patients with a personal history of CKD had a 3.4-fold increased risk of death due to COVID-19 (HR = 3.43, 95% CI 1.07–11.0). The highest risk was observed in immunosuppressed patients (HR = 6.44, 95% CI 1.49–17.8). No other significant associations were observed in the study sample.

## 4. Discussion

The present study analyzed the survival experience among adult inpatients who were diagnosed with PCR-confirmed COVID-19 following the cessation of the global health emergency and during the SARS-CoV-2 endemic era. This research provides valuable insights into the complex interplay between patient characteristics, disease course, and outcomes in this post-emergency phase.

The survival rates, in general, exceeded those documented in the same cohort throughout the wild-type emergency and subsequent pandemic waves [11]. Moreover, even with a hospitalization period extending to 14 days, the computed rates remained higher than 65% (0.66, 95% CI 0.56–0.73).

Interestingly, there were no significant differences in disease outcomes concerning COVID-19 vaccination status, gender, age, or other clinical characteristics that consistently exhibited associations with adverse disease outcomes throughout the pandemic, such as a personal history of type 2 diabetes mellitus [12,13]. The absence of a protective effect from vaccines might be attributed to the extended interval (over 6 months) between the last vaccine administration and the onset of illness, possibly resulting in decreased antibody titers [14]. As for type 2 diabetes mellitus, the absence of an association within the study sample might stem from a potential deficiency in statistical power within the multiple regression model, indicated by the computed *p*-value slightly exceeding the significance threshold (p = 0.056).

The linkage between certain comorbidities and an increased mortality risk was also starkly evident. Participants with a personal history of CKD and immunosuppression exhibited a significantly higher prevalence in the group with fatal outcomes. Similar findings have been previously published [15,16,17,18,19]. These findings underscore the persisting vulnerability of patients with pre-existing health conditions in the post-emergency phase. Collectively, these findings suggest that the post-emergency phase is characterized by a more even distribution of risk factors and that vaccination might not be the sole determinant of survival.

The inclusion of exclusively PCR-confirmed cases represents a significant strength of this study. However, it is essential to acknowledge the potential limitations inherent to the research. Firstly, our analysis was constrained by a limited sample size, a consequence of the low hospitalization rates observed during the post-emergency phase under examination. We focused exclusively on non-ambulatory cases, which exhibited more severe clinical symptoms and, consequently, a higher risk of adverse outcomes. Therefore, conducting larger-scale investigations that also encompass patients with mild symptoms not necessitating hospital admission could potentially yield more comprehensive insights into this area.

Secondly, a notable limitation is the absence of genomic data to ascertain the prevalent SARS-CoV-2 variants. Thirdly, we were unable to measure antibody levels subsequent to SARS-CoV-2 infection or vaccination. Incorporating such measurements could have held clinical and epidemiological value, particularly in assessing the impact of COVID-19 vaccination status on survival rates. Finally, our study did not encompass an analysis of age-stratified data, a methodological omission that curtailed our ability to investigate how various age groups responded to the exposures under scrutiny.

## 5. Conclusions

This study’s findings underscore the persistent gravity of COVID-19, even beyond the global health emergency phase, highlighting the need for targeted interventions for patients with specific comorbidities and vulnerabilities. The complex interplay of patient characteristics, disease severity, and underlying health conditions continues to shape survival outcomes. As the world navigates the post-pandemic landscape, these insights contribute to the informed formulation of healthcare policies and individualized patient management strategies.

## Figures and Tables

**Table 1:** Characteristics of the study sample (*n* = 152) for selected variables, Mexico 2023.

Characteristic	In-Hospital Outcome		*p*	Follow-Up (Days)
	Recovery	Death		
Gender				
Female	75 (59.1)	10 (40.0)	0.079	651
Male	52 (40.9)	15 (60.0)		492
Age (years, median and IQR)	62 (42–76)	68 (59–81)	0.156	1143
COVID-19 vaccination status				
Not vaccinated	101 (79.5)	20 (80.0)	0.973	910
One dose	1 (0.8)	0 (0)		7
Two doses	16 (12.6)	3 (12.0)		129
Three doses	9 (7.1)	2 (8.0)		97
Pneumonia at hospital admission				
No	109 (85.8)	15 (60.0)	0.002	923
Yes	18 (14.2)	10 (40.0)		220
Personal history of:				
Tobacco use, yes	5 (3.9)	3 (12.0)	0.099	71
Obesity (BMI ≥ 30), yes	15 (11.8)	5 (20.0)	0.268	150
Asthma, yes	4 (3.2)	0 (0)	0.369	22
COPD, yes	11 (8.7)	2 (8.0)	0.914	119
Type 2 diabetes mellitus, yes	26 (20.5)	12 (52.0)	0.001	261
Arterial hypertension, yes	38 (29.9)	11 (44.0)	0.169	317
CKD, yes	8 (6.3)	7 (28.0)	0.001	74
Immunosuppression (any cause), yes	2 (1.6)	5 (20.0)	< 0.001	53

Abbreviations: COVID-19, coronavirus disease 2019; IQR, interquartile range; BMI, body mass index; COPD, chronic pulmonary obstructive disease; CKD, chronic kidney disease. Notes: (1) The absolute frequencies (n) and relative frequencies (%) are presented, unless specified as the median. (2) The *p*-values from chi-squared or U-test are reported accordingly.

**Table 2:** Factors associated with survival rates, Mexico 2023.

Characteristic	HR (95% CI), *p*	
	Bivariate Analysis	Multivariate Analysis
Gender		
Female	1.00	1.00
Male	2.10 (0.94–4.69), 0.071	2.79 (0.92–8.49), 0.067
Age (per each additional year)	1.01 (0.99–1.03), 0.276	1.02 (0.99–1.04), 0.236
COVID-19 vaccination status		
Not vaccinated	1.00	1.00
One dose	0.73 (0.16–3.70), 0.685	0.50 (0.11–2.63), 0.482
Two doses	1.11 (0.33–3.81), 0.861	0.59 (0.13–2.66), 0.497
Three doses	0.76 (0.17–3.27), 0.707	0.70 (0.13–3.66), 0.668
Pneumonia at hospital admission		
No	1.00	1.00
Yes	2.38 (1.06–5.37), 0.036	1.97 (0.78–4.99), 0.151
Personal history of:		
Type 2 diabetes mellitus		
No	1.00	1.00
Yes	3.35 (1.52–7.35), 0.003	2.33 (0.93–5.88), 0.056
CKD		
No	1.00	1.00
Yes	5.41 (2.22–13.17), <0.001	3.43 (1.07–11.0), 0.038
Immunosuppression (any cause)		
No	1.00	1.00
Yes	4.39 (1.61–11.96), 0.004	6.44 (1.49–17.8), 0.013

Abbreviations: HR, hazard ratio; CI, confidence interval; COVID-19, coronavirus disease 2019; CKD, chronic kidney disease. Notes: (1) Generalized linear regression models were used to obtain the reported estimates. (2) The estimates from the multiple regression model were adjusted for all the variables presented in the table.

## Data Availability

The data and materials analyzed in this study are available from the corresponding author upon request.
